# How Hybrid Organizations Respond to Institutional Complexity: The Case of Norway

**DOI:** 10.1007/s11266-022-00514-2

**Published:** 2022-08-01

**Authors:** Hilde Svrljuga Sætre

**Affiliations:** Department of Business Administration, Western Norway University of Applied Sciences, Bergen, Norway

**Keywords:** Hybrid organizations, Social enterprise, Institutional logic, Welfare-state regimes

## Abstract

In past decades, hybrid organizations and institutional complexity have received growing attention, yet questions remain about how hybrids manage institutional complexity in the Nordic welfare states. This article investigates how Norwegian social enterprises (SEs), a subset of hybrid organizations, internally manage contradictory demands when externally engaging with multiple logics. The data consists of interviews of leaders and staff members from five SEs, and the findings show that most institutional referents hold a public-sector logic which may crowd out the hybrid nature of SEs. Depending on the conflicting demands, SEs mix decoupling and selective coupling when responding to them. Some were also found to rely on the structural responses of organizational compartmentalization. Compared to the blended hybrids, the structural hybrids experience less internal tension when managing institutional complexity since logic compartmentalization allows the organizations to attend both to their *in-use* logic and *at-play* demands. The data yield compelling insights into how the Nordic welfare state may incite a specific configuration of SE where logic compartmentalization appears as a pragmatic choice.

## Introduction

In recent years, hybrid organizations have received growing research attention. Hybrids can be defined as “organizations that draw on at least two different sectoral paradigms, logics and value systems” (Doherty et al., 2014), thus by nature, they are arenas of contradiction. Hence, they do not fit neatly into the established categories of private, public and voluntary organizations (DiMaggio & Powell, 1983). Rather, they embody multiple institutional logics defined as historically dependent patterns of rules, beliefs, actions, identities, values and material practices and they operate in organizational environments that exert pluralistic and often contradictory demands (Kraatz & Block, 2008). Social enterprise (SE) can be considered a subset of hybrid organizations as they pursue a dual mission: Their activities typically embody a social-welfare logic and a commercial logic as they seek to address societal issues through entrepreneurship (Mair et al., 2015). SEs thus navigate distinct institutional logics and domains. Yet, rather than being driven only by the need to maximize profits for owners and stakeholders, SEs’ surpluses are mainly reinvested in the enterprise or the community in which they operate. The logic embodied in SEs can be considered *in-use* logic which is found in their operations, activities and the governance structure. These hybrid logics are embedded in the self-perception or identity of the organization. However, SEs operate within highly institutionalized environments which creates ambiguity regarding incentives and value dispositions within the SEs. These conflicting logics can be understood as logics *at play*, i.e. logics that are prescribed onto SEs by the institutional environment. Scholarly literature has emphasized that SEs are prone to encounter challenges in the external environment due to their hybridity (Woodside, 2018). SEs are therefore a prime example for studying how hybrids experience and respond to highly institutional environments.

Institutional complexity as a framework has shown that challenges arise when external demands *at play* are internalized by a hybrid, affecting the perceptions, logic and value dispositions *in use* (Greenwood et al., 2011). Recent studies have identified different organizational responses hybrids internally employ when confronted by institutional complexity, showing that some manage to sustain multiple logics, some resort to one dominant logic, some compartmentalize logics, some hybrids thrive, and some even fail (e.g. Pache & Santos, 2013). While these studies have yielded fruitful knowledge, there still lacks knowledge on how hybrids manage institutional complexity in the rather novel study context of the Nordic welfare state.

Pertaining to the theoretically defined Nordic, or social-democratic, welfare regime (Esping-Andersen, 1990), Norway is characterized by a comprehensive public sector with an extensive responsibility for providing universal welfare to its citizens (Pedersen & Kuhnle, 2017: 221). Yet with the turn to New Public Management, certain market-inspired procedures e.g. municipalities contracting out certain services to private providers, have nonetheless manifested themselves. Still, the statist value system is highly engrained in the welfare-state tradition, therefore, despite the gradual increase in market solutions to public welfare since the 1980s, there is still a widespread political consensus in Norway on preserving the state’s responsibility for welfare delivery. Yet, the increase of market-like practices has fostered a political debate labelled the “welfare-profiteer debate” which has reached its culminating point on how much profit (if any) is acceptable for commercial actors operating on public contracts to extract from providing welfare services. The debate pressures non-public organizations to demonstrate distance from market motives. Being an imported organizational form, SEs must therefore adapt to a context with strongly entrenched public-sector traditions amidst a debate fostering distrust in non-public welfare providers. Upholding the notion that SEs embody multiple *in-use* logics, they may have to adapt to additional logics *at play* prescribed by a cumbersome bureaucratic welfare system. Comparatively, the Nordic context is characterized by a highly institutional environment constituted by a strong universal welfare state with its policies on welfare provision, as well as by local authorities that are constrained by public procurement regulations when contracting out with private actors. What remains answered is how hybrids manage the institutional complexity found in the Nordic countries, and whether the Nordic model incites a specific configuration of SEs due to its welfare-state traditions. This article investigates how SEs respond to institutional complexity in Norway and what structural and strategic organizational responses they internalize when externally engaging with multiple logics and demands. Methodically, this article employs rich qualitative interview data from five Norwegian SEs collected from 2018 to 2021.

This article offers valuable empirical and theoretical contributions when exploring how Norwegian hybrids experience and respond to institutional complexity and directs attention to the institutional complexity found in the Nordic countries. Second, it contributes to the theorizing of structural hybrids by demonstrating how hybrids can create organizational compartments in which their distinct *in-use* logic can prevail. Third, and in line with recent work (e.g. Perkmann et al., 2019), the article challenges the assumption that structural hybrids always consist of single-logic compartments, which, is a useful addition to current literature. Finally, it also contributes to theory building within the framework of institutional logics.

In the following, I review the theoretical framework, followed by a description of the research setting. Next, I outline the methodological choices and considerations. Finally, I present the main findings, followed by a discussion and a conclusion.

## Theoretical Framework

Institutional logics are overarching rules and norms shaping the values and goals of an institutional field and make behavior predictable (Thornton et al., 2013). Conceptualized as ideal types, each institutional order distinguishes unique principles and practices that influence organizational behavior (*ibid*.). This framework is a fruitful analytical tool when seeking to understand how organizations (SEs) are influenced by their contexts (institutional environments). In a stylized form, “pure” organizations are aligned with one specific *in-use* logic (Mair et al., 2015). Commercial organizations embody a commercial logic offering services or goods to obtain a financial return to shareholders which are their major stakeholder groups while promoting efficient service delivery (Nicholls, 2010). Voluntary organizations embody a social-welfare logic, addressing wicked problems and prioritizing their beneficiaries, often disadvantaged groups in society, and are characterized by member ownership and revenue generation from donation and member fees (Woodside, 2018). Conversely, hybrids typically embody both in their operations. They address societal problems experienced by disadvantaged groups, while also relying on subsidies by focusing on income strategies (Mair et al., 2015). However, the representation of these ideal-type logics is conditioned by the contexts in which they emerge. As with languages, where the pronunciation of words may vary across geographical locations, we may assume that there also exist different logic *dialects*, with nuanced characteristics. Therefore, SEs will unlikely embody pure social welfare and commercial logic at the same time, but instead *dialects* of them. These logic *dialects* will unlikely be equally fundamental; thus, depending on the aim, structure and goal of the SEs, they operate with two *in-use* logics, a *dominant logic*, while additional logics are *minority logics* (Durand & Jourdan, 2012). However, when faced with demands from actors with the power to evaluate their legitimacy in the field (i.e. institutional referents) that impose conflicting *at-play* logics, tension within the SEs may arise over the prioritizing of goals which can lead to mission drift (Doherty et al., 2014). Upholding the notion that SE is an imported idea that is adopted and adapted into the Nordic context, SEs are submitted to specific demands characteristic of the Nordic institutional environment which likely will affect the SEs prioritization of *in-use* logic.

### Managing Institutional Complexity in Hybrids

The extant research has discovered that when hybrids encounter and manage competing logics *at play*, they select between structural and strategic organizational responses (Pache & Santos, 2013; Perkmann et al., 2019).

One type of response to competing logic can be found in the structure of the hybrid. *Blended hybrids* (alternatively hybrid organizations) represent a single entity embodying multiple *in-use* logic throughout the organizations (Greenwood et al., 2011). This structural solution enables hybrids to exploit different resources that are unattainable by “pure” organizations (Perkmann et al., 2019). Yet, the combination of two (or more) *in-use* logics might create internal tensions since satisfying institutional demands of one logic may require defying demands of another (Greenwood et al., 2011). It can provoke internal tensions among organizational members, create ambiguity in decision-making processes, and, externally, it can create challenges linked to their external legitimacy vis-à-vis institutional referents. *Structural hybrids* are hybrids where different “compartments” of the organization operate according to different logics (Kraatz & Block, 2008). Structural hybrids create structural compartments where the different subunits of the organization operate according to different principles (Perkmann et al., 2019). The compartmentalization enables them to address different audiences and/or deploy different methods. However, while alleviating certain challenges that blended hybrids face, this solution may trigger challenges in integrating the different compartments into the organization, running at risk of organizational fragmentation (Greenwood et al., 2011). While blended hybrids reap the organizational benefits from only one of the sector-dependent legal structures, studies (e.g. Battilana & Dorado, 2010) have documented that SEs have been able to exploit the benefits of two sectors by compartmentalizing, i.e. establish two separate entities. As such, SEs may reconcile competing *at-play* logics with *in-use* logics by carrying out activities expected by institutional referents through one legal entity and another type of activity through the other. Empirically, blended hybrids are expected to experience more internal tensions in meeting with competing *at-play* logics, as institutional referents can push them to dispose of certain logics, compared to structural hybrids which may enact *at-play* logics and *in-use* logics through compartmentalization without receding any of them.

*Decoupling, compromise* and *selective coupling* have in the last decades been exposed as strategic responses in, and in analyses of, hybrids’ encounter with institutional complexity (Pache & Santos, 2013). *Decoupling* entails symbolically adhering to and endorsing practices prescribed by an external *at-play* logic, while at the same time conducting practices promoted by *in-use* logic. In practice, this means that organizations create and uphold a separation between symbolically adopted policies and their organizational behavior (Tilcsik, 2010). This strategy is often adapted to instances where demands prescribed by the external environment conflict with the *in-use* logic of the organization (Pache & Santos, 2013). Thus, hybrids symbolically adapt to these *at-play* demands, while continuing to carry out practices closer to the organizations’ missions. Empirically, decoupling is expected to be found in situations where SEs seek to gain access to grants, i.e. they may symbolically demonstrate compliance, yet in practice carry out activities as usual (Battilana & Dorado, 2010).

*Compromise* relates to the attempt to carry out demands from the logic *at play* in an altered form by finding acceptable balances between the external demand and the *in-use* logics (Oliver, 1991). Compromise entails actual change in behavior, yet the change balances *in-use* and *at-play* logic. Being a less documented strategy, compromise can indeed be a viable option for hybrids to cope with institutional complexity (Kraatz & Block, 2008). In this study, Norwegian SEs may adhere to external *at-play* demands by conforming to the minimum standard by creating a new behavior that merges elements of both types of logic.

Finally, when met with competing demands, SEs may adopt creative configurations of selected practices to meet external demands (Battilana & Dorado, 2010), *Selective coupling* resonates well with the concept of “cultural toolkits” (Swidler, 1986), i.e. responding to various types of contextual issues by employing different configurations to solve them. Such creative mixtures may have elements of symbolic and actual change and can therefore be considered a “catchall” response as it includes a wide variety of actions. Hybrids can secure appraisal of both *in-use* and *at-play* logics as they have access to a broader repertoire of institutional templates, which they can combine. This is called the “Trojan horse” entailing process where so-called “illegitimate” actors adopt and enact the *at-play* logic in the field enabling them to gain acceptance for entering the field (Pache & Santos, 2013). Empirically, SEs considered illegitimate is expected to rely on selective coupling by carrying out activities demanded by the logic *at play* in the institutional environment simultaneously with their main activities and *in-use* logics.

We are now armed with theoretical concepts that help us understand how SEs manage institutional complexity. In the following, I present the study setting.

## Study Setting

Norway is characterized by a large public sector providing universal services to its citizens. Despite a historically long-lived cooperation between the public and nonprofit sector in delivering welfare, commercial welfare production has made itself relevant in all areas of society (Selle et al., 2018). In the provision of welfare, there is a relatively stable set of relationships between the public, private and nonprofit sectors: This constellation is built upon a state that largely produces welfare services itself for buys them through procurement from other, private welfare producers. The relevance of the non-profit sector as a welfare provider has been diminished due to the extent of the public sector, and the slow but steady increase of market mechanisms (Selle et al., 2018). A highly sensitive political question is privatization of welfare. The political left champions public ownership to secure all citizens equal access to services, while the political right champions marketization and privatization to secure the welfare state’s economic sustainability. The question has manifested itself in the heated “welfare-profiteer debate” where examples of commercial welfare producers generating substantial profit have added fuel to the disagreement.

SE emerged in Norway in the early 2000s, and the number of SEs has steadily increased to approximately 300–400 (Eimhjellen & Loga, 2016; Kobro et al., 2017). Since there is no formal organizational form for SEs, they can choose between legal forms from the third and private sector. The extent to which the choice of legal organizational form is caused by ideological orientations or pragmatic adaptions to obtain funding is at present date unclear, but most likely the choice is pragmatic, i.e., they select legal forms based on the probability of attracting funding (Enjolras et al., 2021). The choice of legal form reflects the distribution of responsibilities, risk, taxation, as well as legal rights and duties. It also decides the economic sector affiliation of the SE. In the nonprofit sector, SEs can use organizational forms such as voluntary organizations, associations and cooperatives. In the private sector, SEs can use organizational forms of privately-owned enterprises, e.g. LLCs and *ideal* LLCs. The latter form implies returning any potential profit to the organization, rather than to shareholders. Both LLCs and ideal LLCs are regulated under and abide by the same legislation. Yet, to become an *ideal* LLC, a legal requirement is that the organization declares no personal dividend in the organizational statutes allowing ideal LLCs to call themselves not-for-profit organizations.

Recent studies have suggested that SEs will be difficult to recognize as different from commercial and voluntary organizations as the division of labor between the three economic sectors already are well-established (Enjolras et al., 2021). Moreover, SEs have experienced the need to adapt to other institutional referents’ demands to gain funding (Hauge & Wasvik, 2016). A compelling example of this is the public grant allotted by one of the main institutional referents, the Norwegian Welfare Directorate (NWD), targeting SEs working with inclusion and poverty. This grant is premised on their label as non-profit or not-for-profit (e.g., voluntary organization, associations or ideal LLCs).

## Data and Methods

In the following, I present the methodological choices in this study. A qualitative and exploratory design was used to answer the research question, as this approach is recommended when studying phenomena lacking a well-developed understanding (Yin, 2009). While there does not exist any formal organizational form or registry for SEs in Norway, a mapping of organizations self-identifying as SEs on their websites was conducted. The selection of informants was further based on SEs (1) receiving funding or support from more than one institutional referent; (2) carrying out more than one activity in their operation; (3) with an organizational lifespan of at least five years. This approach is similar to that of purposive sampling (Guest et al., 2017). Prior to the data collection, a dozen SEs were contacted, however, five SEs were recruited to the sample. The sample consists of seven informants. Two informants (staff and founder) were interviewed from SE 1 and 5. The founders of SE 2, 3 and 4 were interviewed once. Due to concerns for anonymity, their names have been omitted. Their governance arrangements are listed in the table below:

**Table:** 

Organization	1	2	3	4	5
Structural solution	Structural hybrid	Structural hybrid	Blended hybrid	Blended hybrid	Blended hybrid
Legal form	Two legal entities: *association* and *ideal LLC*	Two legal entities: *association* and *ideal LLC*	*Ideal LLC*	*Ideal LLC*	*Voluntary organization*
Sector affiliation	Private and third sector	Private and third sector	Private sector	Private sector	Third sector
Governance arrangement	Same person as general manager and chairman of the board in both entities	Both entities share some of the same board members	General manager is kept separate from the board	General manager is also a board manager	Same person as general manager and chairman of the board
Main activity	Sale of products to the public and private sector to employ immigrants	Sale of platform primarily to the public sector to educate adolescents	Sale of courses for young dropouts to the public sector	Sale of products and services to the private and the public sector	Intersectoral collaboration with public sector organizations
Occupational origin of the social entrepreneur	Private sector	Third sector	Private sector	Public and private sector	Public sector

### Limitations

The sample size of SEs in this study is small. During the study, attempts were made to include more SEs, but these refrained from participating due to e.g. covid-19. The sample size of informants is also small. Staff and board members could have informed the analysis about how staff experience institutional complexity within the organizations. However, most of these organizations are small and with few employees, thus the informants selected represent the most vital roles in the organizations. Therefore, while this article contributes to the SE literature, it is nonetheless hard to infer beyond these cases.

### Data Analysis

A thematic analysis (Braun & Clarke, 2019) was applied and carried out. First, an analysis was completed of each separate interview focusing on the experienced *at-play* demands. This informed the study of whether and the degree to which external demands affected the *in-use* logic of the SEs. Second, I identified the conflicting demands and structured them along the following categories: legitimate *legal form* of the organization, legitimate *governance structure, criteria* for funding, and what *activities* SEs should run. Finally, the responses to these demands were analyzed and classified according to the organizational responses. This step of the analysis also enabled a proper identification of the logic in effect. The analysis suggests that SEs naturally embody two *in-use* logics, while pressured to respond to one *at-play* logic. Equally to languages where pronunciation nuances exist in different geographical areas, the logics identified vary from the theoretical ideal types and should therefore not be interpreted *stricto *sensu. Rather, they should be interpreted as logic *dialects*, a metaphor for the empirical representations of the ideal types that are contextually dependent.

The *in-use* logics identified relate to the commercial and social-welfare logics yet represented as *dialects* of them. In support of scholarly literature, the SEs embody two *in-use* logics, namely a *commercial* and a *social-welfare* logic. Yet, different from a “pure” commercial organization selling goods and services to consumers, SEs operating on the commercial logic *dialect* instead emphasize the search for and reliance on subsidies and public contracts. They are thus motivated to find income strategies and employ commercial-like procedures to obtain funding. They sell services and products, primarily through contracts with public authorities, and in few instances, to private enterprises. While they do not operate in a market similar to “pure” commercial organizations, they operate in a quasi-market competing for public contracts. The analysis illustrates that while the SEs may conceive themselves as commercial actors, the environmental pressures they encounter are not primarily competitive pressures. As such, whereas the SEs compete, they are submitted to institutional demands that do not usually characterize a pure commercial logic, hence the logic *dialect*.

The *social-welfare logic dialect* emphasizes cross-sectoral collaboration in the creation and production of welfare. The collaboration’s framework defines their impact area, which often is under the auspices of the public sector. Altruistic actions, e.g. helping others in the local community, secure their legitimacy.

Finally, the identified logic *at play* relates to the public-sector logic dialect characteristic of the Nordic context. This logic dialect is governed by the political economy of the welfare sector. SEs are considered suppliers of cost-efficient services which are obtained through public procurements or other contracts. The political agenda determines the range and duration of the work manifested in the SEs’ contracts with public authorities. Listed in Table 1 below are the logic dialects.

**Table 1:** Logic dialects

	In use logic	In use logic	At play logic
Loeic dialect	Commercial logic	Social-welfare logic	Public-sector logic
Economic system	Private and public subsidies	Non-profit	Welfare capitalism: political economy of the welfare sector
Role of SE	Producer of innovative cost-efficient solutions competing against other suppliers	Partner for social innovation	Supplier on the quasi-market
Nature of work	Selling products/services	Partner in new intersectoral forms of collaboration	Public procurement of short-termed tenders tailored to the public sector
Supplier of conditions	Entrepreneur and subsidy provider	Reach of the collaboration's framework	Social issues on the political agenda
Use of outputs	Increase size to reach a broader target group	Establish networks of new intersectoral forms of collaboration	Modernizing public welfare services to reach demands of the citizens
Evaluation of Legitimacy	Quality of services and public institutions procuring tenders	Altruistic action, helping others in need	Authority-based qualification of efficiency, responsiveness and quality of service
Reward	Economic sustainability, success in service production	Helping the local community, benevolence	Political legitimacy vis-à-vis public authorities

## Findings

In the following, I present the findings of how the SEs manage institutional complexity based on their structural and strategic organizational responses. The main tensions between the SEs and the institutional referents relate to their *legal form*, *governance structure*, public authorities’ *criteria for funding* and the SEs’ *activity*.

All SEs experienced the *at-play* public-sector logic to exert conflicting demands and all internalized enactment to it. First, a pattern emerged between the selection of structural solutions: Structural hybrids were able to attend to their *in-use* logics, while also enact the external *at-play* logic due to logic compartmentalization. Blended hybrids, on the other hand, expressed more inconveniences when encountering *at-play* demands while seeking to adhere to their *in-use logic.* Second, all SEs were found to respond through decoupling and selective coupling. Compromise was not identified. Third, the data reveal that there is a strong field-level consensus regarding the appropriate way to operate: By adhering to the public-sector logic that most institutional referents hold. Since the institutional referents act as gatekeepers of public and private grants, SEs are therefore highly dependent on their acceptance as legitimate actors in the field.

### Legal Form: Same Shit, Just New Wrapping

All but one organization expressed a change in legal status. SE 1 and 2 created two separate entities becoming structural hybrids. This structural response permits them to exploit the benefits of both legal organizational structures. SE 3 and 4 changed their legal status from LLCs to *ideal* LLCs, by declaring no personal dividend in their statutes. These four SEs operate with a dominant commercial-logic dialect, yet the *at-play* demands pressured them to enact the public-sector logic and, consequently, change their legal statuses. SE 5 operates with a dominant social-welfare logic dialect, and its general manager had been supervised by public authorities to organize as a voluntary organization during its start-up phase and has remained a voluntary organization. Indeed, most schemes targeting SEs demand that they are listed in the Voluntary Registry implying that they must be non-profit or not-for-profit separating the organization from pure commercial motives. The founder of SE 4 explained the change in legal status due to external demands from NWD. While the founder publicly advocates for personal dividend, she does not believe it’s possible due to demands from the external environment. SE 4 is registered in the private sector, and in meeting with *at-play* demands, she changed the status to an *ideal* LLC (decoupling), thus conforming to the external pressure. She shared this response with the founder of SE 3. However, both founders expressed that to secure the organizations’ main functions and mission, they kept their organizations in the private sector. Thus, the external environment pressured both founders to create ideal LLCs with statutes prohibiting dividend. The founder of SE 4 explains:

*Only a few months after creating the SE* *as an LLC, I realized that to obtain funding from the NWD my organization had to be registered in the Voluntary Registry, so I had to change the legal form from an LLC to an ideal LLC. I only did this to be eligible for this grant. However, the goal of my organization, the activity and our ideology has not changed. It’s the same shit, just new wrapping (4).*

As the informant underscores, this change is a symbolic adaptation to the demand from institutional referents that do not affect the organization internally. While both the SEs stressed the inconvenience of formally changing the legal status, SE 1 and 2 selected a different strategy for ensuring public funding.

In meeting the same demands from the *at-play* logic, SE 1 established an ideal LLC in addition to her association to secure the organization’s main activity and sources of revenue. SE 2 created an association in addition to his ideal LLC for the same reason. This structural response enables them to uphold their main activities that enhance their sources of revenue in one compartment while enacting and adhering to *at-play* demands in another compartment. The following quote from the founder of SE 2 illustrates this strategy:

*The main enterprise is registered as an association, but I must admit that this is an opportunistic decision. Initially, we wanted to establish an LLC and receive a tax ID number, but the fastest way to do this was by establishing an association. Also, we were aware that our main potential donors demanded a legal form compatible with the Voluntary Registry. But honestly, we do not operate as an association with members [...]. After having scaled up the enterprise, it was important for us also to create an ideal LLC so that we could carry out different activities, receive support from private investors, and commercialize our platform without having to fundamentally change anything internally in the organization (2).*

This suggests that Norwegian hybrids can maneuver around competing demands by combining creative response mixes while continuing to run their activities as usual. The structural hybrids SE 1 and 2 expressed less inconvenience regarding the conflicting *at-play* demand due to the structural response of compartmentalization. As the informant expressed, it is vital for her to have the two compartments to ensure that the organization does not compromise its mission. She also stated that although the governance structure of Norwegian associations should be structured democratically, it is not, implying a symbolic compliance in the formal status, but not internalized in practice. Finally, among the informants, compartmentalization, was characterized as a less inconvenient strategy when met with the competing *at-play* public-sector demands.

#### Governance Structure: Challenging Perceptions of Traditional Voluntary Organizations?

Appropriate governance structure was also found to be a conflicting demand. The appropriate way for SEs to be organized is defined by institutional referents holding the public-sector logic, and interestingly, this demand was only experienced by SE 5, the voluntary organization operating with a dominant social-welfare logic dialect. The founder of SE 5 is both the chairman of the board and the general manager of the organization. When applying for a funding scheme in Municipality X, an inquiry was launched against the founder due to what was labeled an “undemocratic governance structure”. A staff member expressed that the funding scheme does not require any specific type of organizational structure, nor has this question emerged in relation to the other SEs whose governance structures are similar:

[Municipality X] *submitted a complaint against us since our governance structure is undemocratic.* [The founder] *is both chairman of the board and the general manager, and according to *[X] *it* *is not considered best practice. But* [the founder] *wants to secure her and her employees’ salaries, right? She started the organization, she developed the project, she knows the product, therefore she should be the general manager. At the same time, she is the chairman of the board and wants to secure the strategy, concept and activity of the organization.* [... ]. *It is not democratic, but this is an* [SE] *not a traditional voluntary organizatin (*5).

The *at-play* public-sector demand imposed by Municipality X has created tension between the *in-use* logic in SE 5. The founder is now assessing whether to become an ideal LLC allowing her more freedom to structure the organization. Yet, she also wants to secure a productive cooperation and continue ongoing projects with public authorities. This is a compelling example of how institutional complexity can be difficult for hybrids to manage without compromising the organizations’ own missions. Interestingly, in the sample, only SE 3 has separated the general manager from the board of the organization. In the remainder SEs, their general managers are either the chairman or a board member. So, why has only SE 5 been subject to an inquiry? This question is too complicated to be coherently addressed here, but it is an important illustration of how and why the concept of SE can be difficult to adopt and adapt in a Nordic context with a large welfare state with a longstanding tradition of member-based voluntary organizations with few to no commercial motives. While historically such organizations have been vital in the establishment of the welfare state, they are rarely viewed as compatible with commercial-like motives.

### Criteria for Funding

Another conflicting *at-play* demand relates to criteria for funding. Here, the SEs have had a unison response: satisfying symbolic concern. To receive public funding from most (public) funding schemes such as the NWD, the activity of an SE must include or activate voluntarism. All informants expressed having experienced pressure to incorporate voluntarism in their organizations. However, all, save SE 5, asserted that although it is an important factor separating SEs from “pure” commercial organizations, voluntarism is sometimes loosely defined in applications and often used only symbolically to obtain funding. One informant from a structural hybrid expressed:

*There is not always much voluntarism to be found in the activities of SEs. I mean, we do have some voluntary actors in our enterprise, but my experience is that SEs must state that they have incorporated some type of voluntarism to receive public funding*. *To be honest, there is not much voluntarism in our operations. Our values are focused on helping our target group, not to ensure that we can arrange for a tea party with two volunteers each week* (1)*.*

While not faking compliance with the *at-play* public-sector demand, the informant suggests that SEs strategically include voluntarism in their operations to receive funding from institutional referents. Symbolically adhering to this demand may be considered a pragmatic choice when their practices and activities conflict with external, *at-play* demands. The other structural hybrid, SE 2, voiced the same concern and stated, “Voluntarism should not be a formal criterion as it does not define whether we do a good job, or not” (2). This indicates that SEs are conscious in their communication with institutional referents and use deliberate wording depending on funding criteria.

Next, jargon related to funding applications was also found to be a specific *at-play* demand resolved by the SEs through decoupling. Public funding schemes relate to social issues on the political agenda, and public authorities want non-public actors to tailor tenders to or apply for project contracts on issues the public sector wants addressed. One social issue SEs are asked to address is integration. However, the data show that public funding schemes premised on SEs also define how this integration should be carried out. The blended hybrid, SE 5, working with integration, had its project proposal rejected by ‘Municipality X’ due to wrong terminology:

*Two years ago, we wrote an application to a budget item named ´Inclusion of Immigrants’. Apparently, we overused the word ‘integration’ in the application, and it was rejected. When we changed the word 'integration' to the word 'inclusion', we received the funding from the exact same budget item.* *In reality, neither the way we operated, nor the application changed. It really depends on what wording we use in the applications, as you can see, there are strings attached* (5).

Again, decoupling emerges as a pragmatic response. Rather than altering the activity or operation in the application proposal, the strategic response was to alter how the application is written.

### Activity: Counter-Productive Juggling

Finally, the SEs are also submitted to pressures from the *at-play* public-sector logic regarding specific activities. The SEs with the dominant *in-use* commercial logic dialect, incorporated combinations of commercial activities (i.e. sale of services and products) with project activities carried out for or in collaboration with public services. This supports the extant research suggesting that hybrids reconcile *in-use* and *at-play* logics by enacting a combination of activities drawn from different logics to secure funding and endorsement from a wider range of actors (Pache & Santos, 2010). Yet, this time-consuming juggling may also have detrimental effects such as mission drift, i.e. sacrificing *in use* organizational goals to fulfill demands *at play*. While none of the informants expressed having experienced mission drift, they all voiced how *at-play* demands could be detrimental to their SEs’ goals. This is especially the case when the SEs operating on a dominant commercial logic are forced to carry out short-termed projects for the public sector to secure endorsement and legitimacy in the field, while at the same time carrying out their main activity or working on acquiring long-termed contracts with public authorities. The blended hybrids 3 and 4, expressed that juggling activities contradictory to the SEs’ mission, yet vital for its survival and legitimacy, was exhausting. The founder of SE 3 stated that she had to carry out specific activities to secure funding while at the same time highlighting the unpredictability of such assistance schemes:

*The funding we receive from public assistance schemes demand a project activity. So, to gain access to funding, we must do these projects while at the same time carrying out our main work. We juggle different types of activities at once. Yet, these assistance schemes are unpredictable and their budgets are low. Additionally, we don’t know if we will receive the same funding the next year. We are therefore afraid to hire people* (3)*.*

While juggling different activities was expressed as tedious and in conflict with the SE’s main *in-use* objective, it also points to the uncertainty of these schemes. The informant further highlighted that these assistance schemes come with ‘strings attached’. Still, they are vital for the SE’s legitimacy vis-à-vis institutional referents. SE 4 underscored the same concerns:

*We have done a couple of small ‘stunts’ which have been essential for the survival of the organization*. *Last summer we did a summer activity for* [the target group], *which an association gave us a small sum for, but it is not exactly business* […]. *This goes to show that the SE ecosystem is fragmented and complex, and that it is difficult to get someone to fund our activities* [...]. *Yet, we are all completely dependent on writing these funding applications *(4)

The blended hybrids 3 and 4 responded to these *at-play* demands by selective coupling, i.e. creatively combining activities demanded by referents with a public-sector logic with the SEs’ main activities. Interestingly, the founder of the structural hybrid, SE 1, expressed having previously been dependent on adherence to this counter-productive juggling. But, since the organizational compartmentalization, the SE now has a sustainable economy due to its commercial platform anchored in the ideal LLC. The other structural hybrid also managed to scale up the SE’s range due to its commercial strategies yet continued to be dependent on applying to assistance schemes. A staff member expressed her concern regarding detrimental effects of the dependence on funding schemes:

*I believe that it is counter-productive to spend many hours each year writing funding applications. Of course, we do it, but we waste our time writing these applications, rather than focusing on our main objective. You know, the public funding schemes are short-term, and often for no more than a year at a time. It is quite exhaustive to apply because we must wait six months before we receive an answer* (2).

The results show that the continuous sequence of writing and applying for funding to secure endorsement from a wide range of actors is imperative for Norwegian hybrids to survive. The data also suggest that when meeting *at-play* demands of the public-sector logic dialect, SEs are likely to tone down certain *in-use* objectives as legitimacy vis-à-vis institutional referents is vital. Interestingly, however, the structural hybrid, SE 1, managed to become independent of short-term projects. While more profound research is needed, a rising curiosity is whether compartmentalization is an optimal hybridization strategy in a context with a dominant public sector. Seemingly, SE 1 can secure mission compliance, adhere to external demands and gain legitimacy.

**Fig. 1:**
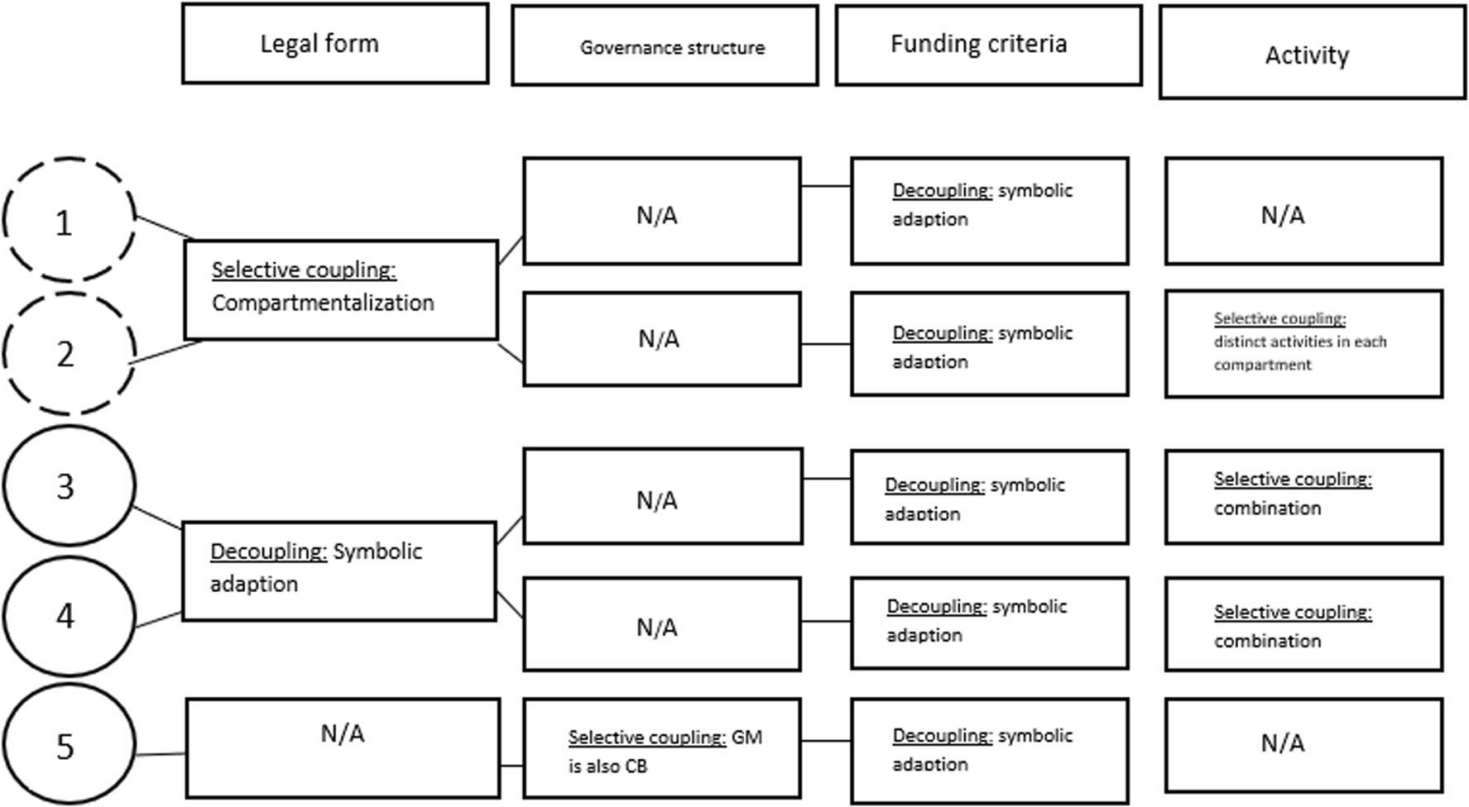
Response patterns

Figure 1 sums up the response patterns of the SEs. The SEs with a broken line represent the structural hybrids, and full circle, blended hybrids.

## Discussion

This article seeks to contribute to the understanding of how hybrid organizations (SEs) respond to institutional complexity in a Nordic welfare state with a large public sector responsible for providing universal services to its citizens. The article attempts to explain how SEs adapt to the highly institutional environment constituted by the Nordic welfare model. More specifically, it studies how SEs adapt to conflicting demands from institutional referents and how this affects them. It also investigates whether the Nordic context incites a specific configuration of SEs. In doing so, the article explores the structural and strategic organizational responses SEs employ when externally engaging with external *at-play* logics. The study shows that the public-sector logic dialect is the dominant *at-play* logic in the institutional environment which the SEs must enact to gain funding and legitimacy in the field. This pressures SEs to defy their *in-use* commercial logic dialect.

### Favoring the ‘Toolkit Approach’

An important insight from this study is that SEs face conflicting *at-play* demands when applying for funding and when seeking to gain legitimacy in the field. Additionally, the SEs highlight that assistant schemes are their most vital sources of income. In the context of the Nordic model, the natural role of the public sector appears to be evaluating SEs’ legitimacy through the distribution of schemes and contracts. Regardless of the consequences, it has for the *in-use* logics of the SEs, they must enact the *at-play* public-sector logic dialect. The data illustrates that the external environment commands SEs, especially operating with a dominant *in-use* commercial logic, to behave more like non-profit organization, thus deviating from certain commercial strategies. This appears to create tensions in the SEs’ self-perception of being social *and* commercial actors. While not completely abandoning their commercial strategies, they are pressured to emphasize their social mission. Nevertheless, although the highly institutionalized environment in Norway affects these SEs, the empiricism also demonstrates that the SEs can select different strategies to secure compliance with their *in-use* logic while also enacting *at-play* demands to gain legitimacy in the field. Since none of the SEs responded by seeking compromise, it might suggest that SEs’ role in the welfare mix is not influential enough to negotiate or put pressure on the institutional referents. This finding reflects indeed a small and fragmented SE field in Norway, but it also highlights the dominance of the public sector as a welfare provider.

The interviews further revealed that the SEs respond to the *at-play* demands by mixing different structural and strategic organizational responses to the conflicting demands. This strategy resembles that of “cultural toolkits” (Tracey et al., 2011), i.e. employing different sets of configurations when met with different types of issues. Indeed, the hybridity of SEs can be favorable in that it secures them access to different institutional templates to solve the organizational tensions that they meet. This may help them create an organizational configuration combining elements of the *at-play* demands while adhering to *in-use* logic. Additionally, it may also help them obtain a wider support range. When mixing the strategic responses selective coupling and decoupling, the SEs do not blindly comply with the *at-play* demands. Rather, the informants reflect on the contradictory demands prescribed by institutional referents and express contrafactual perceptions (sometimes even internal resistance) although, in the end, complying with the demands.

### Toward a Nordic Configuration?

Another valuable insight is how the two types of SEs, blended and structural hybrids, experience and respond differently to institutional complexity. The blended hybrids expressed more inconvenience with the demands from the external logic. This is especially the case for the SEs operating with a dominant commercial-logic dialect. The informants from SE 1, 2, 3 and 4 stressed how vital it was for the organizations’ survival to comply with the *at-play* demands of the public sector. SE 2 and 3, enacted practices demanded by the public-sector logic dialect despite the negative consequences it had for their operations. SEs are incipient organizations in Norway, and due to a culminating point of the welfare-profiteer debate, they may be looked at askance by the public sector. Adopting behaviors prescribed by the *at-play* public-sector logic dialect can give “illegitimate” actors legitimacy and acceptance for entering the field. As seen, however, this can also be experienced as troublesome since the SEs might have to deviate from *in-use* logic and value dispositions. Although none of the SEs experienced mission drift per se, the founders of SE 3 and 4 expressed how the organizational goals were negatively affected by conflicting *at-play* demands. As such, the institutional environment seems to crowd out the SEs hybridity as the dominant commercial-logic dialect must be toned down, while their minority social-welfare logic dialect is considered legitimate and may prevail.

SE 5 with organizational ties to the third sector, expressed being pressured into mimicking the practices of the public sector to secure a productive cooperation and continued funding. Public authorities questioned the lack of democratic governance when the founder of SE 5 arranged the organization different from a traditional voluntary organization. This may indicate that institutional referents impose specific expectations on non-profit, voluntary organizations in Norway, as opposed to e.g. ideal LLCs. The case being that cross-sectoral collaborations are under the auspices of the public sector this may suggest that public authorities can demand more from SEs organized as voluntary organizations, like SE 5.

Finally, the structural hybrids, SE 1 and 2, managed to attract a broader funding base as both organizations receive subsidies from public and private actors. They use different compartments to apply for different funding and contracts. While this strategy has been observed prior (Battilana & Dorado, 2010), it is still quite unusual for hybrids, especially for organizations tied to the private sector to establish an additional organization tied to the third sector to access grants. However, by compartmentalizing distinct logics into different organizations pertaining to different economic sectors, this strategy ensures that neither *in-use* logics are compromised by external *at-play* demands. It can also enable them to become sustainable as SE 1 expressed. Finally, the SEs may ensure their legitimacy in the external environment by appealing to a variety of institutional referents. Thus, the question that remains is whether creating compartments is a pragmatic SE configuration in the Nordic context due to the prominent role of the public sector?

### Contextual Implications

With a strong state and large public-welfare system, public authorities are responsible for identifying social issues that need to be addressed, defining how to address them, and evaluating which actors that may solve them. While this institutional environment may negatively affect the nature of hybrids, public authorities do constitute the most vital institutional referents that SEs depend on as public authorities are gatekeepers of important schemes and evaluators of their legitimacy in the field. Regarding the four conflicting demands (legal form, governance structure, criteria for funding and activity) all SEs have at some point enacted and responded to the *at-play* public-sector logic. However, in doing so, the *in-use* commercial-logic dialect, becomes subordinate in these instances, especially for the SEs with a dominant commercial-logic dialect. This supports recent findings (Enjolras et al., 2021) suggesting that the hybrid nature of SEs may be crowded out by a strong state and well-established third and private sector organizations.

Furthermore, although a rather unusual strategy for SEs, logic compartmentalization may be a pragmatic solution, especially for SEs tied to the private sector. By compartmentalizing, SEs may avoid certain *at-play* demands prescribed by institutional referents. Also, compartmentalization may allow organizations to easier attend to their mission while at the same time adhere to external demands. Although this indication remains to be thoroughly investigated, this configuration of SEs could be relevant and advantageous in the Nordic context as structural hybrids may both broaden their sources of income, adhere to *in-use* and *at-play* logic and gain legitimacy in the field, all at the same time.

## Conclusion

This article has explored how hybrids (SEs) respond to institutional complexity. It has also discussed whether the context of the Nordic welfare state incites a specific configuration of SEs. The article illustrates how the Nordic welfare state, with a large public sector responsible for providing universal welfare to its citizens, affects how SEs operate and engage with institutional referents. The public-sector logic *dialect* is identified as the prevailing *at-play* logic that all SEs respond to and enact by decoupling or selective coupling. The study context illustrates a highly institutionalized environment in which institutional referents operate with the *at-play* public-sector logic dialect. Additionally, they act as gatekeepers of funding schemes and evaluators of the SEs’ legitimacy. Norwegian SEs are therefore highly dependent on their acceptance in the field. The data suggest that logic compartmentalization might be a pragmatic choice in the Nordic countries as it allows SEs to attend to the *in-use* logics, thus not risking mission drift, and adhere to external *at-play* demands. Yet, further research is needed before any firm conclusion can be drawn.
